# Report of a Case of Ligation of Both Common Carotids for Secondary Hemorrhage

**Published:** 1864-09

**Authors:** Russell Murdoch

**Affiliations:** Assistant Surgeon P. A. C. S.


ART. VIII.—*Report of a Case of Ligation of both Common Carotids for Secondary Hemorrhage.* By RUSSELL MURDOCH, Assistant Surgeon P. A. C. S.

Benjamin Creicy, private company "F," 42d Virginia infantry, entered Winder Hospital, May 9th, 1863, on account of a gun-shot wound passing through the larynx, at the upper margin of the thyroid cartilage, on both sides, and impinging slightly upon the thyro-hyoid membrane.

On May 12th, about two o'clock, A. M., I was called, during the absence of the physician who had charge of the case, to check hemorrhage from the wound. I found him in an exsanguined condition, about thirty ounces of blood on the floor and still bleeding profusely; extreme dyspnœa and distressing nausea. Hemorrhage was temporarily checked by pressure on the main vessel of the right side until assistance could be called, and a rapid consultation held. It was considered impracticable to tie the bleeding ends of the artery, because of the tumid condition of the surrounding parts, the impossibility of administering chloroform, and the too obscure light furnished by a tallow candle with which to make the necessary dissection. The common carotid was the artery selected for ligature. The operation was performed at the seat of election. It was rendered extremely difficult by the collateral circumstances above mentioned, along with the violent struggles of the patient.

The case progressed favorably until the morning of the 15th, when, at a similar unseasonable hour, I was again called to him, on account of hemorrhage from the left side. The circumstances being the same, the other common carotid was tied. In both these hemorrhages, the bleeding was as active towards the pharynx as externally. Much blood was swallowed, producing extreme nausea; the larynx also got its full share, as indicated by the violent efforts to get breath. For the following thirty-five hours, no symptoms of an untoward character ensued; the circulation was to all appearances adequate for the nutrition of the parts to be supplied; the functions of the brain perfect, and the wound healthy. Unfortunately, on the 20th, a hemorrhage ensued, which terminated in death.

An *autopsy* threw some light upon the sources and sequence of the different hemorrhages. On the proximal side of both the ligatures there was found the ordinary plug of lymph; on the distal of the right, the vessels were flaccid, and appeared smaller than those of the left, while the latter were of their natural calibre and appearance. The superior thyroid artery was traced past the wound, while the hyoid branch approached it. This vessel, on the right, was lost in the ulcerated and engorged tissues, while on the left, although the vessel was for a part of its course lost, yet an open-mouthed artery was discovered in its course, opening directly back into the pharynx, which was, without doubt, the source of the fatal, if not of the two last hemorrhages. The brain was not examined, for want of proper instruments.

The difficulty of tracing the vessels, even during the favorable conditions offered by a *post-mortem* examination, produced by the effused and ulcerated conditions of the tissues and the depth of the vessel which was found, fully convinces us that the course adopted, although the result was unfavorable, was the only one of which the circumstances admitted.

